# Impact of Preoperative Biliary Stenting on Intestinal Dysfunction and Perioperative Complications After Pylorus-Preserving Pancreaticoduodenectomy

**DOI:** 10.3390/medicina61030391

**Published:** 2025-02-24

**Authors:** Gelu Mihai Breaza, Florin Emil Hut, Octavian Cretu, Simona-Alina Abu-Awwad, Ahmed Abu-Awwad, Laurentiu Sima, Radu Gheorghe Dan, Cristina Ana-Maria Dan, Raluca Maria Closca, Flavia Zara

**Affiliations:** 1 Doctoral School, “Victor Babes” University of Medicine and Pharmacy, 300041 Timisoara, Romania; gelu.breaza@umft.ro; 2 University Clinic of Surgery I, “Victor Babes” University of Medicine and Pharmacy, 300041 Timisoara, Romania; octavian.cretu@umft.ro (O.C.); sima.laurentiu@umft.ro (L.S.); radu.dan@umft.ro (R.G.D.); cristina.dan@umft.ro (C.A.-M.D.); 3 Center for Hepato-Bilio-Pancreatic Surgery, “Victor Babes” University of Medicine and Pharmacy, 300041 Timisoara, Romania; 4 Ist Clinic of Obstetrics and Gynecology, “Pius Brinzeu” County Clinical Emergency Hospital, 300723 Timisoara, Romania; alina.abuawwad@umft.ro; 5 Department of Obstetrics and Gynecology, Faculty of Medicine, “Victor Babes” University of Medicine and Pharmacy, 300041 Timisoara, Romania; 6 Department XV—Discipline of Orthopedics—Traumatology, “Victor Babes” University of Medicine and Pharmacy, 300041 Timisoara, Romania; ahm.abuawwad@umft.ro; 7 Research Center University Professor Doctor Teodor Sora, “Victor Babes” University of Medicine and Pharmacy, 300041 Timisoara, Romania; 8 Department of Pathology, Emergency City Hospital, 300254 Timisoara, Romania; raluca.moaca@umft.ro (R.M.C.); flavia.zara@umft.ro (F.Z.); 9 Department of Microscopic Morphology, University of Medicine and Pharmacy “Victor Babes”, 300041 Timisoara, Romania

**Keywords:** preoperative biliary stenting, intestinal dysfunction, pancreaticoduodenectomy, perioperative complications, delayed gastric emptying, pancreatic fistula

## Abstract

*Background and Objectives*: Preoperative biliary stenting (PBS) is commonly used to manage obstructive jaundice in patients undergoing pylorus-preserving pancreaticoduodenectomy (PPPD). However, the impact of PBS on intestinal barrier function and perioperative complications remains controversial. This study aims to evaluate the effect of PBS on intestinal dysfunction and surgical outcomes, focusing on the influence of the stent duration. *Materials and Methods*: In this prospective cohort study, 235 patients undergoing PPPD for resectable pancreatic neoplasms at Timișoara Municipal Emergency Clinical Hospital (2016–2024) were analyzed. Patients were divided into two groups: those with PBS (n = 98) and without PBS (n = 137). Intestinal barrier function was assessed pre- and postoperatively using biomarkers such as zonulin, fecal calprotectin, and serum lipopolysaccharides (LPS). Perioperative outcomes, including pancreatic fistula, delayed gastric emptying (DGE), infections, and hospital stay, were compared. Additionally, outcomes were stratified based on stent duration (2–3 weeks vs. 3–4 weeks). *Results*: PBS was associated with significantly higher levels of zonulin, fecal calprotectin, and serum LPS postoperatively, indicating compromised intestinal barrier function. The stented group had a higher incidence of pancreatic fistulas (Grade B/C: 27.5% vs. 13.1%, *p* < 0.01), DGE (25.5% vs. 13.1%, *p* = 0.008), postoperative infections (34.7% vs. 17.5%, *p* = 0.002), and prolonged hospital stay (16.9 ± 4.2 days vs. 14.5 ± 3.7 days, *p* = 0.019). Prolonged stenting (3–4 weeks) was associated with worse outcomes compared to shorter stenting durations (2–3 weeks), including increased rates of infections, sepsis, and ICU stay (*p* < 0.05 for all comparisons). *Conclusions*: Preoperative biliary stenting is associated with increased intestinal barrier dysfunction, systemic inflammation, and higher rates of perioperative complications following PPPD. Prolonged stenting durations (>3 weeks) further exacerbate these risks. Limiting the PBS duration to 2–3 weeks, alongside optimized perioperative management, may help reduce postoperative morbidity and improve surgical outcomes.

## 1. Introduction

Pancreatic cancer is a highly aggressive malignancy, with surgical resection remaining the only curative option [1]. Pylorus-preserving pancreaticoduodenectomy (PPPD) is commonly performed for resectable pancreatic neoplasms, aiming to improve postoperative nutritional outcomes and maintain gastrointestinal function [2]. However, many patients with obstructive jaundice due to pancreatic tumors require preoperative biliary stenting (PBS) to alleviate bile duct obstruction and improve hepatic function before surgery [3]. Despite its benefits, PBS has been associated with an increased risk of perioperative complications, including infections, delayed gastric emptying (DGE), and pancreatic fistula formation [4].

An indwelling biliary stent can promote bacterial colonization and systemic inflammation, potentially exacerbating surgical complexity and prolonging recovery [5]. Studies suggest prolonged stent placement may impair the intestinal barrier function, leading to increased permeability, bacterial translocation, and postoperative infections [6,7]. Biomarkers such as zonulin, fecal calprotectin, and serum lipopolysaccharides (LPS) have been used to assess the gut integrity in patients undergoing major abdominal surgery, offering insights into the systemic effects of biliary stenting [8].

Furthermore, the duration of stent placement before surgery has emerged as a critical factor influencing perioperative outcomes. Prolonged stenting (>4 weeks) has been linked to a higher incidence of complications such as pancreatic fistula, intra-abdominal infections, and prolonged hospital stays. In contrast, shorter stenting durations (≤4 weeks) may reduce these risks but require the careful timing of surgery to ensure optimal biliary decompression [9].

Despite the existing literature, there remains a gap in understanding the comprehensive impact of preoperative biliary stenting on intestinal dysfunction and perioperative complications following PPPD. The relationship between stent duration, intestinal barrier integrity, and clinical outcomes has yet to be fully elucidated.

This study aims to evaluate the impact of preoperative biliary stenting on perioperative outcomes in patients undergoing pylorus-preserving pancreaticoduodenectomy (PPPD), focusing on several key aspects. First, it seeks to assess intestinal barrier dysfunction by measuring pre- and postoperative biomarkers, including zonulin, fecal calprotectin, and serum lipopolysaccharides (LPS), to determine the effect of stenting on gut permeability. Second, the study aims to analyze perioperative complications by comparing the incidence of pancreatic fistula, delayed gastric emptying, infectious complications, and length of hospital stay between stented and non-stented patients. Third, it evaluates the impact of the stent duration by comparing clinical outcomes in patients with stenting durations of 2–3 weeks versus those with 3–4 weeks, investigating whether a longer duration is associated with an increased risk of postoperative morbidity. Lastly, the study aims to provide clinical recommendations to optimize the management of patients requiring biliary stenting before PPPD, ultimately aiming to minimize complications and improve surgical outcomes.

By addressing these objectives, this study seeks to explore the intricate relationship between preoperative biliary interventions, intestinal health, and surgical outcomes in patients with pancreatic neoplasms.

## 2. Materials and Methods

### 2.1. Study Design and Patient Selection

This prospective cohort study was conducted to evaluate the impact of preoperative biliary stenting on intestinal dysfunction and perioperative complications in patients undergoing pylorus-preserving pancreaticoduodenectomy (PPPD) for pancreatic neoplasms. The study was carried out between 2016 and 2024 at Timișoara Municipal Emergency Clinical Hospital, with data prospectively collected from institutional medical records. Patients were divided into two groups: those who underwent preoperative biliary stenting (stented group) and those who did not (non-stented group). Further stratification within the stented group was performed based on stenting duration into two subgroups: 2–3 weeks and 3–4 weeks.

A total of 235 patients who underwent pylorus-preserving pancreaticoduodenectomy (PPPD) for resectable pancreatic neoplasms were prospectively included in the study, conducted between 2016 and 2024 at Timișoara Municipal Emergency Clinical Hospital. Patients were categorized into two groups based on preoperative biliary stenting status. Group A consisted of 98 patients (41.7%) who underwent preoperative biliary stenting, while Group B included 137 patients (58.3%) who did not receive stenting before surgery. Within the stented group, patients were further stratified based on stent duration into two subgroups, 2–3 weeks and 3–4 weeks, to evaluate the potential impact of prolonged stent placement on perioperative outcomes.

Tumor resectability was determined according to the criteria established by the National Comprehensive Cancer Network [10] and the International Study Group of Pancreatic Surgery [11]. Advanced preoperative imaging techniques, including contrast-enhanced computed tomography with vascular reconstruction as the primary modality, were used for assessment. In selected cases, additional imaging, such as magnetic resonance imaging and endoscopic ultrasound, was performed to evaluate tumor involvement further and optimize surgical planning.

All surgical procedures were performed by multiple surgical teams, with composition evolving over the study period. However, to maintain consistency, all surgeons involved had extensive experience in pancreatic surgery, with a minimum of 10 years of practice and full proficiency in performing PPPD. The surgical technique was standardized across all cases, strictly adhering to established principles, including meticulous tumor resection and digestive tract reconstruction techniques.

Perioperative and postoperative management protocols were applied uniformly across the study population, including standardized antibiotic prophylaxis, nutritional support, pain management, and complication surveillance. These protocols were periodically updated under emerging clinical guidelines and best practices to ensure optimal patient care.

### 2.2. Data Collection

Clinical and demographic data, including age, sex, BMI, and comorbidities, were obtained from patient records to characterize the study population. Tumor features were documented to assess disease severity, including size, stage, and histological type. Preoperative biliary stenting (PBS) was analyzed based on the use of plastic stents only, with an insertion method exclusively via ERCP and a duration categorized as 2–3 weeks vs. 3–4 weeks.

Intestinal barrier dysfunction was assessed using specific biomarkers (zonulin, fecal calprotectin, and serum lipopolysaccharides), clinical evaluation (gastrointestinal symptoms, postoperative ileus, delayed gastric emptying, and infectious complications), and the lactulose/mannitol ratio as a marker of intestinal permeability. Inflammatory markers, including CRP, NLR, serum albumin, and total bilirubin, were recorded preoperatively and postoperatively to monitor systemic inflammation and liver function.

Perioperative outcomes were documented, such as blood loss, operative time, and transfusion requirements. The primary outcomes included postoperative complications (pancreatic fistula, delayed gastric emptying, and infections), while secondary outcomes covered hospital and ICU stay duration, 30-day readmission, and mortality rates. All postoperative infections were microbiologically confirmed, as this was a required criterion for diagnosis and targeted antibiotic treatment.

The impact of stent duration on complications was analyzed, alongside the relationship between inflammatory markers, gut dysfunction, and surgical outcomes, providing insights to optimize patient management.

### 2.3. Inclusion and Exclusion Criteria

Inclusion criteria are as follows:

- Patients aged ≥18 years diagnosed with resectable pancreatic neoplasms;
- Patients undergoing pylorus-preserving pancreaticoduodenectomy (PPPD) with curative intent;
- Patients with histopathologically confirmed ductal adenocarcinoma of the pancreatic head, eligible for PPPD;
- Availability of complete preoperative and postoperative clinical and biochemical data, including inflammatory and intestinal permeability biomarkers;
- Patients with a documented preoperative biliary stenting period of 2–3 weeks or 3–4 weeks, confirmed by imaging or procedural records;
- Hemodynamically stable patients, eligible for major pancreatic surgery;
- Patients without contraindications to surgery based on preoperative cardiac, pulmonary, and hepatic function assessments;
- No prior history of major abdominal surgeries affecting gastrointestinal function;
- Patients who provided informed consent for participation in the study and agreed to follow-up evaluations;
- Patients with preoperative bilirubin levels reduced to acceptable surgical limits post-stenting.

Exclusion criteria are as follows:

- Chronic gastrointestinal disorders that could interfere with the assessment of intestinal barrier function, such as inflammatory bowel disease (Crohn’s disease, and ulcerative colitis) or celiac disease;
- Use of immunosuppressive therapy (e.g., corticosteroids, chemotherapy, or biologic agents) within six months prior to surgery, which could alter inflammatory marker levels and postoperative recovery;
- Active systemic infections, including viral hepatitis, tuberculosis, or HIV, which may confound the evaluation of surgical outcomes and systemic inflammation;
- History of alcohol abuse or substance dependency, which may affect liver function, nutritional status, and perioperative recovery;
- Prolonged preoperative hospitalization exceeding four weeks due to unrelated medical conditions, leading to potential deconditioning and increased surgical risk;
- Patients with uncorrected coagulation disorders, such as hemophilia or thrombocytopenia, that could increase the risk of perioperative bleeding and complications;
- Participation in other clinical trials evaluating experimental surgical techniques or pharmacological interventions that could confound study outcomes;
- Uncontrolled psychiatric disorders, including severe depression or psychotic disorders, that could impact compliance with postoperative care and follow-up;
- Malnutrition unresponsive to optimization, with persistent cachexia or severe weight loss (>15% body weight in 3 months), making surgical intervention high-risk;
- Pregnancy or lactation, due to the ethical concerns and potential complications associated with major abdominal surgery during these physiological states.

### 2.4. Surgical Technique

Preoperative biliary stenting is a procedure used to relieve bile duct obstruction in patients with pancreatic head tumors or other conditions that cause biliary obstruction before undergoing surgery, such as the modified Whipple procedure. This intervention involves the insertion of a stent—a small, flexible tube—into the bile duct to facilitate bile flow from the liver to the intestines, preventing complications such as jaundice, cholangitis, and liver dysfunction. Preoperative stenting can be performed endoscopically via endoscopic retrograde cholangiopancreatography (ERCP) or percutaneously through percutaneous transhepatic biliary drainage (PTBD). The primary goal of this approach is to improve liver function and overall patient condition before surgery, potentially reducing perioperative complications. However, its routine use remains a topic of debate, as some studies suggest that preoperative stenting may increase the risk of postoperative infections and complications, such as pancreatitis or stent-related occlusion. Therefore, the decision to perform preoperative biliary stenting should be individualized based on the patient’s clinical status, the severity of biliary obstruction, and the anticipated timing of surgery [12,13,14].

All patients included in this study underwent the modified Whipple procedure, also known as pylorus-preserving pancreaticoduodenectomy. This complex and extensive surgical technique is designed to remove tumors located in the head of the pancreas while preserving key anatomical structures to maintain the digestive system’s functionality as much as possible. The procedure involves the meticulous excision of the pancreatic head, which is the primary tumor site. In addition to this, several surrounding structures are also removed to ensure thorough elimination of malignant tissue. These structures include the entire duodenum, the first segment of the small intestine, part of the proximal jejunum, the subsequent portion of the small intestine, the gallbladder, and a section of the bile duct. This comprehensive surgical approach is crucial to reduce the likelihood of residual cancer cells and enhance postoperative recovery.

A distinctive feature of this modified technique is the preservation of the pylorus, the muscular valve that regulates the passage of food from the stomach into the small intestine. Unlike the standard Whipple procedure, which involves resecting the distal stomach, the pylorus-preserving variant retains the stomach’s continuity, aiming to decrease complications such as delayed gastric emptying and support improved postoperative nutritional outcomes.

Following the resection phase, reconstructing the digestive tract is essential for restoring normal digestive processes. This is accomplished through a series of precise surgical anastomoses, including an end-to-side pancreaticojejunostomy, which links the remaining pancreas to the jejunum to facilitate the flow of pancreatic enzymes; a choledochojejunostomy, which connects the bile duct to the jejunum to reestablish bile drainage; and a gastrojejunostomy, which directly attaches the stomach to the jejunum, bypassing the excised duodenum. These intricate reconstructions ensure the digestive system’s continuity despite removing multiple critical structures [15,16,17].

### 2.5. Statistical Analysis

The statistical analysis in this study was performed using GraphPad Prism 6. Descriptive statistics were utilized to summarize the baseline characteristics of the study population, including measures of central tendency and dispersion. Continuous variables, such as age, body mass index (BMI), serum albumin levels, and total bilirubin, were reported as mean ± standard deviation (SD) to understand the data distribution within each group. Categorical variables, including comorbidities such as diabetes and hypertension, were expressed as absolute numbers and percentages, allowing for straightforward comparisons between groups.

The independent-samples *t*-test was employed to compare continuous variables between the stented and non-stented groups. This test was chosen to determine whether there were significant differences in key preoperative and postoperative parameters, such as serum biomarkers (e.g., hemoglobin and C-reactive protein) and perioperative outcomes (e.g., operative time and intraoperative blood loss). A significance level of *p* < 0.05 was considered statistically significant for all comparisons.

For categorical variables, including the incidence of complications such as pancreatic fistula, delayed gastric emptying (DGE), and postoperative infections, the chi-square (χ^2^) test was applied to assess differences in proportions between the stented and non-stented groups. In cases where expected frequencies were low (typically <5 in any cell), Fisher’s exact test was utilized to ensure the robustness of statistical comparisons. Additionally, subgroup analyses were conducted within the stented group to evaluate the impact of different stenting durations (2–3 weeks vs. 3–4 weeks) on postoperative outcomes. The same statistical methods, including independent-samples *t*-tests for continuous variables and chi-square tests for categorical data, were applied to identify potential differences between these subgroups.

All statistical analyses were performed with a 95% confidence interval (CI), ensuring that the findings were interpreted with an appropriate degree of certainty. The data were visualized using bar charts and scatter plots generated in GraphPad Prism 6 to facilitate the interpretation of key trends and differences between groups.

### 2.6. Ethical Consideration

This research adhered to the ethical principles established by the Declaration of Helsinki. Before initiating data collection, approval was secured from the Institutional Review Board (IRB) of Timisoara Municipal Emergency Clinical Hospital (Nr.225/2 September 2015). Due to the study’s retrospective design, the IRB granted a waiver for informed consent; nonetheless, strict measures were taken to uphold patient confidentiality. All collected data were anonymized to safeguard privacy and prevent any potential identification of individuals. As the study was based solely on the analysis of pre-existing medical records, without any direct interventions or patient involvement, there were no associated risks to participants.

## 3. Results

Table 1 presents the demographic and clinical characteristics of the patients included in the study, comparing those who underwent preoperative biliary stenting (n = 98) with non-stented patients (n = 137). The two groups were comparable in terms of age, sex distribution, body mass index (BMI), and the prevalence of pre-existing diabetes mellitus and hypertension (*p* > 0.05). However, a significantly higher proportion of smokers was observed in the stented group compared to the non-stented group. Laboratory parameters revealed that the stented group had significantly lower serum albumin levels and higher total bilirubin levels, indicating a more pronounced impairment of liver function. Additionally, the stented group exhibited substantially lower hemoglobin levels, reduced lymphocyte counts, and an elevated neutrophil-to-lymphocyte ratio, suggesting a higher degree of systemic inflammation. C-reactive protein (CRP) levels were also significantly higher in the stented group, further supporting the presence of an inflammatory response. Perioperative outcomes demonstrated more extended hospital stays, prolonged operative time, and more significant intraoperative blood loss in the stented group. Moreover, the need for perioperative transfusions was significantly higher in patients who underwent preoperative biliary stenting. The Charlson Comorbidity Index was slightly higher in the stented group, indicating a higher burden of comorbidities. These findings suggest that preoperative biliary stenting is associated with a more compromised clinical status and increased perioperative risks.

Table 2 presents the comparative analysis of preoperative and postoperative intestinal barrier function and associated complications. Preoperatively, patients in the stented group exhibited significantly higher levels of zonulin, fecal calprotectin, and serum lipopolysaccharides (LPS), suggesting increased intestinal permeability and systemic inflammation. The lactulose/mannitol ratio, a key indicator of gut barrier integrity, was also significantly elevated in the stented group, further indicating compromised gut function in these patients.

Postoperatively (Day 5), intestinal barrier dysfunction persisted and worsened in the stented group, with significantly higher zonulin levels, fecal calprotectin, and serum LPS levels. The lactulose/mannitol ratio remained elevated considerably in the stented group, indicating ongoing intestinal barrier compromise after surgery. Regarding postoperative complications related to intestinal dysfunction, the incidence of postoperative ileus was significantly higher in the stented group than the non-stented group, with a longer duration of ileus observed in stented patients. The occurrence of delayed gastric emptying (DGE, Grade B/C) was also significantly more frequent in the stented group, prolonging recovery and increasing the need for nutritional support.

Furthermore, bacterial translocation, evidenced by positive blood cultures, was more common among stented patients, indicating a higher risk of systemic infection. The incidence of abdominal sepsis was also significantly greater in the stented group compared to the non-stented group, further emphasizing the clinical impact of impaired gut barrier function.

The perioperative complications observed in the stented and non-stented groups reveal significant differences between the two cohorts (as summarized in Table 3). Patients who underwent preoperative biliary stenting had a higher incidence of clinically relevant pancreatic fistulas (Grade B/C), delayed gastric emptying (DGE), and postoperative ileus, all showing statistically significant differences compared to the non-stented group. Additionally, stented patients experienced a notably higher occurrence of intra-abdominal infections and sepsis, suggesting a potential association between biliary stenting and an increased risk of infectious complications.

Surgical complications, including anastomotic leakage, hemorrhage requiring reoperation, and liver dysfunction, were also more frequent in the stented group, indicating a more challenging postoperative course. The length of stay in both the intensive care unit (ICU) and the hospital was significantly longer in the stented group, reflecting the increased burden of complications and prolonged recovery associated with biliary stenting. Furthermore, the 30-day readmission rate was significantly higher among stented patients, reinforcing the need for closer postoperative monitoring and tailored management strategies in this subgroup. Although the 30-day mortality rate was higher in the stented group, the difference did not reach statistical significance.

The perioperative complications observed in patients with preoperative biliary stenting durations of 2–3 weeks versus 3–4 weeks highlight the impact of prolonged stenting on surgical outcomes. As shown in Table 4, patients with extended stent placement experienced a significantly higher incidence of clinically relevant pancreatic fistulas (Grade B/C) and delayed gastric emptying (DGE), with a greater severity of these complications. Moreover, the group with longer stenting durations had notably higher rates of postoperative ileus, intra-abdominal infections, and sepsis, indicating an increased risk of both systemic and localized infections.

Surgical complications such as bile leakage, wound infections, pneumonia, and hemorrhage requiring reoperation were significantly more frequent in the more extended stenting group, indicating a potential adverse effect of extended biliary drainage on postoperative recovery. Organ dysfunction, including acute kidney injury and liver dysfunction, was also more prevalent in patients with stent placement exceeding three weeks, further complicating their perioperative course.

Moreover, patients in the 3–4 weeks stenting group experienced a significantly more extended stay in both the intensive care unit (ICU) and the hospital, reflecting the cumulative impact of increased postoperative morbidity. The 30-day readmission rate was also significantly higher in this group, emphasizing the need for closer follow-up and potentially more intensive postoperative care. Although the 30-day mortality rate was higher among patients with prolonged stenting, the difference did not reach statistical significance.

These findings suggest that prolonged preoperative biliary stenting (3–4 weeks) is associated with a higher risk of perioperative complications, particularly infectious, gastrointestinal, and organ dysfunction outcomes. Optimizing the timing of surgery and minimizing unnecessary stenting duration may help mitigate these risks and improve patient outcomes.

## 4. Discussion

This study explores the impact of preoperative biliary stenting (PBS) on intestinal dysfunction and perioperative complications in patients undergoing pylorus-preserving pancreaticoduodenectomy (PPPD). Our results demonstrate a clear association between PBS and an increased incidence of postoperative complications, including pancreatic fistulas, delayed gastric emptying (DGE), and infections. Furthermore, our analysis provides novel insights by evaluating shorter stenting intervals, highlighting that 3–4 weeks is associated with a significantly higher risk of complications than stenting for 2–3 weeks. These findings contribute valuable information to the existing literature, which predominantly focuses on the impact of long-term stenting durations (>4 weeks) [18,19].

PBS directly impacts the body’s defense barriers, particularly the intestinal barrier, through a series of pathophysiological processes that contribute to increased complications. Biliary stasis induced by a stent alters the normal bile flow to the intestine, leading to dysbiosis by disrupting the intestinal microbiota composition. This promotes the growth of pathogenic bacteria while reducing the production of beneficial metabolites, such as short-chain fatty acids (SCFAs), essential for maintaining intestinal barrier integrity. Dysbiosis creates a pro-inflammatory environment at the local level, exacerbating the epithelial barrier dysfunction [20,21,22].

Our findings, which reveal elevated levels of biomarkers such as zonulin, fecal calprotectin, and serum lipopolysaccharides (LPS), are consistent with previous studies that have demonstrated the adverse effects of biliary stasis on the intestinal microbiota [23,24,25]. Zonulin, a key marker of intestinal permeability, indicates epithelial barrier dysfunction when elevated [26,27]. One study reported that high levels of zonulin are associated with bacterial translocation and systemic inflammation, explaining the increased risk of postoperative complications [28]. Additionally, LPS, a component of Gram-negative bacterial cell walls, activates Toll-like receptors (TLR4), triggering a systemic inflammatory cascade, as demonstrated by Kim et al. [29]. In our stented group, elevated CRP levels further confirm this systemic inflammation, which promotes infectious complications, delays tissue healing, and increases the incidence of pancreatic fistulas.

Another critical mechanism is the formation of bacterial biofilms on the surface of biliary stents, which play a central role in chronic bacterial colonization. Biofilms serve as a constant reservoir of pathogenic bacteria, facilitating the continuous release of toxins into the biliary and intestinal systems. As reported in the literature, this aggravates dysbiosis and local inflammation, significantly increasing the risk of cholangitis and intra-abdominal abscesses [30].

Our findings expand upon this evidence, showing that the pathophysiological mechanisms induced by PBS—from intestinal dysbiosis and bacterial translocation to systemic inflammation and biofilm formation—are interconnected and play a major role in exacerbating postoperative complications. These results underscore the importance of carefully managing PBS, notably by limiting the stenting duration, to minimize its adverse effects on the microbiota, systemic inflammation, and the risk of severe complications.

The persistent inflammation caused by bacterial biofilms on biliary stents significantly contributes to increased intestinal permeability, facilitating bacterial translocation and elevating the risk of sepsis. This mechanism is supported by studies, highlighting that prolonged stent usage worsens systemic inflammation by amplifying biofilm activity, explaining the heightened risk of severe complications, including intra-abdominal infections and sepsis [5,30,31,32]. Our observations align with these findings, as we noted a higher incidence of these complications in the stented group compared to the non-stented group, particularly with prolonged stenting durations (3–4 weeks).

Bacterial biofilms not only amplify systemic inflammation but also impair postoperative healing processes. Bacterial translocation and endotoxemia intensify pro-inflammatory activity, delaying connective tissue formation and proper wound healing. This mechanism likely explains the higher incidence of clinically relevant pancreatic fistulas (Grade B/C) and other anastomotic complications observed in the prolonged PBS group, consistent with some findings, who correlated systemic inflammation with increased rates of these complications [33].

Our study also revealed significantly lower serum albumin levels in patients with PBS, reflecting a compromised nutritional profile. These findings align with other research, who demonstrated that chronic inflammation induced by stenting suppresses hepatic albumin synthesis while increasing inflammatory markers like CRP, contributing to hypoalbuminemia. This physiological imbalance reduces patients’ nutritional reserves, making them less capable of tolerating surgical stress [34,35].

Furthermore, chronic inflammation exacerbates the catabolic state of the body, depleting available nutritional resources and heightening patients’ vulnerability to postoperative complications [36]. Several studies have shown that low serum albumin levels are associated with an increased risk of pancreatic fistulas and anastomotic failures, emphasizing the need for early nutritional optimization before surgery [37,38]. Our results, consistent with this literature, underscore the importance of limiting the stenting duration and implementing rigorous management strategies to mitigate PBS-related adverse effects on patients’ overall health and surgical outcomes.

While the risks of prolonged stenting (>4 weeks) are well-documented, our study introduces a novel perspective by demonstrating that even shorter stenting durations (3–4 weeks) are associated with significantly higher complication rates compared to 2–3 weeks. For example, the incidence of clinically relevant pancreatic fistulas (Grade B/C) was 25.0% in the prolonged stenting group compared to 16.7% in the shorter stenting group, with a similarly higher prevalence of DGE and intra-abdominal infections in the former. These findings suggest a cumulative risk with each additional week of stenting.

In line with previous studies, PBS has been associated with an increased incidence of infectious complications such as cholangitis and intra-abdominal abscesses. However, our detailed analysis of short stenting intervals provides additional insights, showing that bacterial translocation and elevated LPS levels play a central role in the pathogenesis of these complications, underscoring the need to limit the PBS duration to reduce the associated risks [39,40,41].

Our findings are consistent with the previous meta-analyses and randomized controlled trials that have examined the impact of preoperative biliary stenting (PBS) on postoperative outcomes. For instance, a meta-analysis by Scheufele et al. [3] reported an increased incidence of overall complications (odds ratio: 1.40; 95% confidence interval: 1.14–1.72; *p* = 0.002) and wound infections (odds ratio: 1.94; 95% confidence interval: 1.48–2.53; *p* < 0.00001) in patients receiving PBS compared to those who proceeded directly to surgery. Similarly, Gong et al. found that PBS significantly increased the incidence of postoperative morbidity, delayed gastric emptying, and wound infection in patients undergoing pancreaticoduodenectomy [39]. These studies reinforce the association between PBS and increased postoperative complications, aligning with the observations in our cohort.

### 4.1. Clinical Implications, Study Limitations, and Future Research Directions

These findings have significant clinical implications, highlighting the need for clear strategies to optimize the management of patients undergoing PBS before PPPD. First, the PBS duration should be limited to 2–3 weeks whenever possible, with careful surgical planning to mitigate the risks associated with prolonged stenting. Additionally, patients should be evaluated for nutritional deficiencies and provided with preoperative dietary support, including protein supplements and fat-soluble vitamins, to counteract the adverse effects of biliary stasis and systemic inflammation. Simultaneously, strict antibiotic prophylaxis protocols and inflammatory biomarker monitoring are essential for the early identification of high-risk patients. Emerging biomarkers such as zonulin and LPS could also be predictive tools for perioperative complications, enabling personalized therapeutic interventions.

This study has several strengths, including its prospective design, detailed analysis of short stenting intervals (2–3 weeks vs. 3–4 weeks), and the evaluation of specific biomarkers (zonulin, LPS, and fecal calprotectin) to assess the impact of PBS on intestinal dysfunction and perioperative outcomes. Additionally, the use of standardized surgical protocols ensured data consistency and result validity.

However, there are several limitations that should be acknowledged. First, this is a single-center study, which may limit the generalizability of the findings. Second, the study lacks a microbiome analysis, which could have provided further insights into the mechanisms of gut dysbiosis and bacterial translocation following PBS. Third, the relatively small number of patients in certain subgroups may affect the robustness of some conclusions. Additionally, a dedicated multivariate analysis to determine whether PBS is an independent predictor of pancreatic fistula was not performed, and this should be addressed in future research.

Another important limitation is that the exact mechanism underlying the association between PBS and increased DGE incidence remains unclear. Factors such as bacterial translocation, systemic inflammation, and prolonged ICU stay may all contribute to this phenomenon. Further prospective, multicenter studies incorporating microbiome analysis and advanced inflammatory profiling are needed to elucidate these mechanisms better and refine clinical decision-making.

### 4.2. Clinical Implications and Surgical Relevance

The findings of this study highlight important considerations for surgical practice regarding preoperative biliary stenting in patients undergoing pancreatoduodenectomy. Our results suggest that PBS is associated with a higher incidence of delayed gastric emptying and infectious complications, which may be linked to bacterial translocation and systemic inflammation. Given these risks, the decision to perform PBS should be carefully weighed against its potential benefits, particularly in patients without clear indications for biliary decompression.

From a surgical perspective, the meticulous intraoperative handling of the pancreatic remnant, early identification of high-risk patients, and optimized perioperative management may help mitigate the adverse effects associated with PBS. Further studies are needed to refine the patient selection criteria and develop strategies to minimize postoperative morbidity in this population.

## 5. Conclusions

This study demonstrates that preoperative biliary stenting in patients undergoing pylorus-preserving pancreaticoduodenectomy is associated with a higher incidence of postoperative complications, including pancreatic fistula, delayed gastric emptying, and infections. Patients with preoperative biliary stenting exhibited increased systemic inflammation, lower serum albumin levels, and impaired gut barrier function, as indicated by the elevated zonulin, fecal calprotectin, and serum lipopolysaccharide levels. Notably, prolonged stenting durations (3–4 weeks) were linked to worse outcomes, such as a higher incidence of pancreatic fistulas and extended hospital stays, compared to shorter stenting periods (2–3 weeks). These findings highlight the importance of optimizing the timing of surgery, with shorter stenting durations associated with better clinical outcomes. Our results suggest that limiting the preoperative biliary stenting duration to 2–3 weeks, implementing strict infection control protocols, and improving the preoperative nutritional status may help mitigate risks and enhance surgical outcomes. Despite the study’s strengths and prospective design, limitations such as the single-center setting and the lack of microbiome analysis warrant further research. Future multicenter studies are needed to validate these findings and develop optimized management strategies for patients requiring preoperative biliary decompression before pylorus-preserving pancreaticoduodenectomy.

## Figures and Tables

**Table 1:** Demographic and clinical characteristics of the included patients.

Characteristic	Stented Group (n = 98)	Non-Stented Group (n = 137)	*p*-Value
Age (mean ± SD, years)	65.1 ± 8.5	64.2 ± 9.1	0.432
Sex (M/F)	55/43	70/67	0.581
Body Mass Index (BMI, kg/m^2^)	23.8 ± 3.4	24.3 ± 3.5	0.102
Smokers (%)	40 (40.8%)	38 (27.7%)	0.033 *
Pre-existing diabetes mellitus (%)	32 (32.7%)	40 (29.2%)	0.564
Hypertension (%)	50 (51.0%)	58 (42.3%)	0.187
Serum albumin (g/dL)	3.1 ± 0.5	3.5 ± 0.4	<0.001 *
Total bilirubin (mg/dL)	13.1 ± 3.0	4.3 ± 1.4	<0.001 *
Hemoglobin (g/dL)	11.5 ± 1.6	12.0 ± 1.5	0.011 *
Lymphocytes (% of total WBC)	16.2 ± 4.0	19.5 ± 4.8	<0.001 *
Neutrophil-to-lymphocyte ratio (NLR)	4.8 ± 1.7	3.1 ± 1.3	<0.001 *
CRP (mg/L)	47.5 ± 21.8	24.0 ± 12.5	<0.001 *
Length of hospital stay (days)	16.8 ± 4.2	14.6 ± 3.8	<0.001 *
Operative time (minutes)	290 ± 50	280 ± 45	0.045 *
Intraoperative blood loss (mL)	810 ± 225	720 ± 200	<0.001 *
Need for perioperative transfusion (%)	46 (46.9%)	42 (30.7%)	0.021 *
Charlson Comorbidity Index	6.1 ± 1.1	5.8 ± 1.0	0.049 *

* Statistically significant *p*-value.

**Table 2:** Preoperative and postoperative intestinal barrier dysfunction.

Parameter	Stented Group (n = 98)	Non-Stented Group (n = 137)	*p*-Value
Preoperative Measurements			
Zonulin (ng/mL)	70.8 ± 9.5	50.2 ± 8.7	<0.001 *
Fecal calprotectin (µg/g)	318 ± 82	215 ± 70	<0.001 *
Lactulose/mannitol ratio	0.13 ± 0.03	0.09 ± 0.02	0.015 *
Serum lipopolysaccharide (LPS, EU/mL)	56.9 ± 11.8	43.3 ± 9.8	<0.001 *
Postoperative Measurements (Day 5)			
Zonulin (ng/mL)	83.4 ± 10.7	57.1 ± 9.3	<0.001 *
Fecal calprotectin (µg/g)	365 ± 90	250 ± 78	<0.001 *
Lactulose/mannitol ratio	0.16 ± 0.04	0.11 ± 0.03	0.028 *
Serum lipopolysaccharide (LPS, EU/mL)	63.8 ± 13.9	47.5 ± 10.9	<0.001 *
Postoperative complications related to intestinal dysfunction (%)			
Postoperative ileus (%)	30 (30.6%)	20 (14.6%)	0.004 *
Delayed gastric emptying (DGE, Grade B/C)	25 (25.5%)	18 (13.1%)	0.008 *
Bacterial translocation (positive blood cultures)	16 (16.3%)	9 (6.6%)	0.015 *
Abdominal sepsis (%)	12 (12.2%)	6 (4.4%)	0.022 *
Length of postoperative ileus (days)	5.1 ± 1.7	3.0 ± 1.4	0.019 *

* Statistically significant *p*-value.

**Table 3:** Perioperative complications.

Complication	Stented Group (n = 98)	Non-Stented Group (n = 137)	*p*-Value
Pancreatic fistula (ISGPS classification)			
- Grade A	16 (16.3%)	12 (8.8%)	0.048 *
- Grade B	20 (20.4%)	15 (10.9%)	0.027 *
- Grade C	7 (7.1%)	3 (2.2%)	0.041 *
Delayed gastric emptying (DGE)			
- Grade A	24 (24.5%)	19 (13.9%)	0.034 *
- Grade B	14 (14.3%)	9 (6.6%)	0.038 *
- Grade C	9 (9.2%)	5 (3.6%)	0.049 *
Postoperative ileus (%)	30 (30.6%)	20 (14.6%)	0.003 *
Intra-abdominal infections (%)	34 (34.7%)	24 (17.5%)	0.002 *
Sepsis (%)	9 (9.2%)	5 (3.6%)	0.047 *
Bile leakage (%)	10 (10.2%)	7 (5.1%)	0.093
Wound infection (%)	22 (22.4%)	16 (11.7%)	0.026 *
Anastomotic leakage (%)	9 (9.2%)	5 (3.6%)	0.041 *
Hemorrhage requiring reoperation (%)	7 (7.1%)	4 (2.9%)	0.047 *
Liver dysfunction (%)	15 (15.3%)	8 (5.8%)	0.014 *
Necrotizing pancreatitis (%)	5 (5.1%)	2 (1.5%)	0.049 *
Length of ICU stay (days, mean ± SD)	6.5 ± 2.1	4.4 ± 1.8	<0.001 *
Length of hospital stay (days, mean ± SD)	16.9 ± 4.2	14.5 ± 3.7	0.019 *
30-day readmission rate (%)	22 (22.4%)	15 (10.9%)	0.017 *
30-day mortality (%)	8 (8.2%)	5 (3.6%)	0.112

* Statistically significant *p*-value.

**Table 4:** Relationship between stenting duration and postoperative complications.

Complication	2–3 Weeks Stenting (n = 54)	3–4 Weeks Stenting (n = 44)	*p*-Value
Pancreatic fistula (ISGPS classification)			
- Grade A	6 (11.1%)	9 (20.5%)	0.048 *
- Grade B	9 (16.7%)	11 (25.0%)	0.042 *
- Grade C	3 (5.6%)	5 (11.4%)	0.089
Delayed gastric emptying (DGE)			
- Grade A	8 (14.8%)	10 (22.7%)	0.055
- Grade B	5 (9.3%)	8 (18.2%)	0.048 *
- Grade C	3 (5.6%)	6 (13.6%)	0.033 *
Postoperative ileus (%)	11 (20.4%)	16 (36.4%)	0.031 *
Intra-abdominal infections (%)	13 (24.1%)	18 (40.9%)	0.026 *
Sepsis (%)	3 (5.6%)	7 (15.9%)	0.041 *
Bile leakage (%)	3 (5.6%)	7 (15.9%)	0.037 *
Wound infection (%)	8 (14.8%)	12 (27.3%)	0.049 *
Pneumonia (%)	6 (11.1%)	10 (22.7%)	0.048 *
Anastomotic leakage (%)	3 (5.6%)	5 (11.4%)	0.078
Hemorrhage requiring reoperation (%)	2 (3.7%)	5 (11.4%)	0.047 *
Acute kidney injury (%)	5 (9.3%)	9 (20.5%)	0.034 *
Liver dysfunction (%)	4 (7.4%)	10 (22.7%)	0.016 *
Length of ICU stay (days, mean ± SD)	5.2 ± 2.0	8.1 ± 3.1	<0.001 *
Length of hospital stay (days, mean ± SD)	15.1 ± 3.6	19.4 ± 4.4	<0.001 *
30-day readmission rate (%)	8 (14.8%)	14 (31.8%)	0.029 *
30-day mortality (%)	3 (5.6%)	5 (11.4%)	0.112

* Statistically significant *p*-value.

## Data Availability

The datasets used and analyzed during the current study are available from the first author.
